# Emerging Role of Endosomal Toll-Like Receptors in Rheumatoid Arthritis

**DOI:** 10.3389/fimmu.2014.00001

**Published:** 2014-01-16

**Authors:** Ryan Thwaites, Giselle Chamberlain, Sandra Sacre

**Affiliations:** ^1^Brighton and Sussex Medical School, Trafford Centre, University of Sussex, Brighton, UK

**Keywords:** rheumatoid arthritis, toll-like receptor, inflammation, autoimmunity, endosomal toll-like receptors, autoimmunity models, therapeutics

## Abstract

Toll-like receptors (TLRs) and their downstream signaling pathways have been comprehensively characterized in innate immunity. In addition to this function, these receptors have also been suggested to be involved in the pathogenesis of many autoimmune diseases, including rheumatoid arthritis (RA). Murine *in vivo* models and human *in vitro* tissue models of RA have provided a wealth of information on the potential activity of TLRs and components of the downstream signaling pathways. Whilst most early work investigated the cell surface TLRs, more recently the focus has moved to the endosomal TLRs 3, 7, 8, and 9. These receptors recognize self and foreign double-stranded RNA and single-stranded RNA and DNA. The development of therapeutics to inhibit the endosomal TLRs or components of their signaling cascades may represent a way to target inflammation upstream of cytokine production. This may allow for greater specificity than existing therapies including cytokine blockade. Here, we review the current information suggesting a role for the endosomal TLRs in RA pathogenesis and the efforts to target these receptors therapeutically.

## Toll-Like Receptors

Toll-like receptors (TLRs) are a family of evolutionarily conserved pattern recognition receptors. There are 10 human TLRs, which recognize pathogen associated molecular patterns from fungi, bacteria, and viruses, such as lipopolysaccharide (LPS), flagellin, and dsRNA (1). In addition to pathogens, TLRs also respond to damage associated molecular patterns (DAMPs), which are endogenous molecules released at sites of inflammation and tissue damage; examples include High Mobility Group Box 1 (HMGB-1) and Tenascin C (2–4). Most TLRs are expressed at the cell surface where they recognize mainly bacterial products, whereas TLRs 3, 7, 8, and 9 are localized to endosomal compartments and respond to self and foreign nucleic acid structures. TLR3 recognizes dsRNA, whilst TLR7 and TLR8 both respond to ssRNA (5). TLR9 recognizes DNA, the binding event of which was believed to be sequence specific, favoring CpG-rich DNA. However, recent evidence suggests that DNA conformation is actually the key mediator of TLR9 activation, not the DNA sequence (6). Localization of these TLRs to the endosome likely represents a method of sequestering these molecules from inadvertent activation by host nucleic acids.

**Figure 1:**
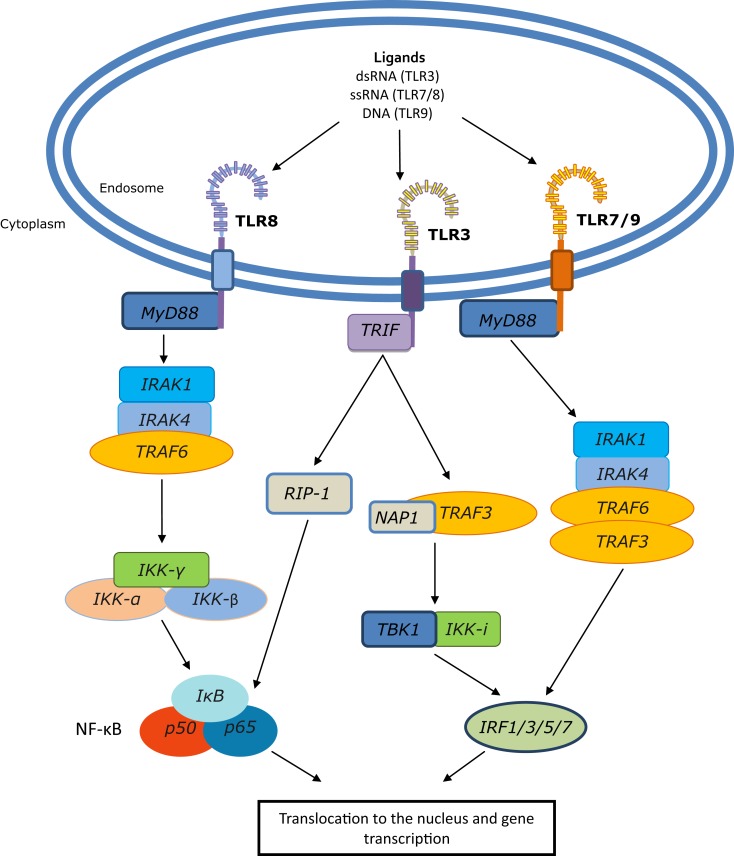
**Endosomal TLR signaling to NF-κB and IRFs**. Following ligand binding, TLRs 7, 8, and 9 engage MyD88; for TLR8 this leads to activation of NF-κB, whereas TLRs 7 and 9 engage IRAK1/4, TRAF3, and TRAF6 to activate IRF family members. TLR3 uses the TLR adaptor TRIF, which can induce NF-κB through the TRAF6 complex and can signal to RIP-1 which, like the TRAF6 complex, can also activate the IKK-α,β,γ complex. This results in dissociation of IκB from NF-κB, which can then translocate to the nucleus and initiate gene transcription. Additionally, TRIF can signal through TRAF3, TBK1, and IKK-i to initiate IRF-mediated transcription.

Toll-like receptors form homo- or hetero-dimers upon activation and ligand binding. This dimerization event brings the TIR domains into close proximity, allowing for the association of adaptor molecules that trigger downstream signaling events (Figure 1). There are four main adaptor proteins; Myeloid differentiation primary response 88 (MyD88), MyD88 adaptor like (MAL), TIR domain containing adaptor inducing interferon-β (TRIF), and TIR domain containing adaptor molecule 2 (TRAM). MyD88 is engaged by all of the TLRs except TLR3, MAL is used by TLRs 2 and 4, TRIF by TLRs 3 and 4, and TRAM by TLR4. The adaptor molecules trigger signaling via either the MyD88 dependent pathway, which activates NF-κB, producing cytokines such as tumor necrosis factor (TNF) and IL-1, or the MyD88 independent pathway that leads to the activation of interferon regulatory factor (IRF) family members and production of type-I interferons (IFN) (7).

The TLRs have been extensively studied in both infectious and chronic inflammatory diseases. In particular, their ability to respond to endogenous ligands has made them attractive candidates in the maintenance of inflammation in many autoimmune diseases, including rheumatoid arthritis (RA).

## Rheumatoid Arthritis

Rheumatoid arthritis is an autoimmune disease affecting approximately 1% of the worldwide population, characterized by the infiltration and accumulation of activated immune cells in the synovial joints. Subsequent chronic production of pro-inflammatory cytokines and matrix metalloproteases (MMPs) leads to destruction of the joint architecture, severe disability, and a reduced life expectancy due to co-morbid complications such as cardiovascular disease (8).

Many different cell populations in the RA joint secrete cytokines and other inflammatory mediators, which contribute to pathogenesis. Macrophages are considered to be a major effector of synovitis, secreting cytokines including TNF, IL-1, and IL-6 (9). This inflammatory environment supports Th17 cell differentiation and suppresses differentiation of regulatory T-lymphocytes, thereby further promoting inflammation. In addition, RA synovial fibroblasts (RASF) secrete IL-6 when stimulated by inflammatory cytokines such as TNF and IL-17 (secreted by Th17 cells), thus perpetuating the inflammatory cycle (10). RASFs can also express receptor activator of nuclear factor κB ligand (RANKL), facilitating differentiation of monocytes/macrophages into osteoclasts, thereby promoting pathological bone erosion (11). B-lymphocytes and their progeny also contribute to the progression of RA pathogenesis through pro-inflammatory cytokine production, in addition to generation of autoantibodies characteristic of disease. Although much is understood about the inflammatory environment in RA, the ligand(s) responsible for sustaining cytokine production in this setting remains unknown.

Conventional treatment involves the use of disease-modifying anti-rheumatic drugs (DMARDs), most commonly methotrexate (MTX), as well as non-steroidal anti-inflammatory drugs, antimalarials, and corticosteroids (12). However, treatment of RA has been greatly enhanced over the past decade by the development of biological therapies targeting molecules implicated in RA pathogenesis. The longest standing are those targeting TNF; the anti-TNF antibodies infliximab, adalimumab, and golimumab, the polyethylene glycol-linked mAb fragment certolizumab pegol, and the soluble TNF receptor-2-IgG-Fc fusion protein etanercept (13, 14). Additional targets of clinically approved biological therapies include the IL-1 receptor (anakinra), the IL-6 receptor (tocilizumab), CD20 on B-lymphocytes (rituximab), and cytotoxic T-lymphocyte-associated antigen 4 (CTLA-4) (abatacept). Other agents in early-stage clinical development include the anti-IL-17 antibodies secukinumab and ixekizumab and the IL-17 receptor-blocking antibody brodalumab (14, 15).

Tumor necrosis factor inhibitors remain the gold standard of biological therapies for RA even though approximately 30% of patients show no significant improvement (16). There is data suggesting that the use of biologics early in the course of disease may induce clinical remission in some patients (15, 17), although many patients do not achieve permanent remission. Some patients also become refractory to treatment with time, due to the production of antibodies against anti-TNF biologicals (18); in this situation tocilizumab has been shown to be a successful alternative (19).

Interestingly, some anti-TNF patients report no clinical improvement in their symptoms but do show a decreased progression of joint damage radiographically (20). This may suggest a different threshold for the inflammatory versus the bone damage-activating effects of TNF. In agreement with this concept, in the human TNF transgenic murine model of arthritis, low-dose administration of adalimumab had no effect on synovial inflammation but significantly reduced bone erosions by inhibiting osteoclastogenesis (21).

Biological therapies, although a clear improvement over DMARDs, do not represent an ideal therapeutic. They require parenteral administration, either intravenously or by subcutaneous injection and are expensive to manufacture with their cost severely restricting use. Global suppression of cytokine function also holds the potential for serious adverse effects such as bacterial infections, invasive fungal infections, and in the case of anti-TNF reactivation of latent tuberculosis (22). In an effort to address these limitations, low molecular weight inhibitors are in development to target intracellular signaling molecules and prevent cellular responses to cytokines. Several small-molecule inhibitors of the Janus kinase family are currently in clinical development for the treatment of RA (23). Tofacitinib, an inhibitor of JAK3 and JAK1 and, to a lesser extent, JAK2 has recently been approved for the treatment of adults with active RA (24).

Small molecular weight inhibitors of inflammation are highly desirable due to their low cost of production. However, targeting downstream signaling molecules may prove to be troublesome due to the ubiquitous nature of many of these molecules in multiple signaling pathways. This has been a particular problem for inhibitors of p38 mitogen-activated protein kinases, which are too toxic for use in the treatment of chronic conditions such as RA (25). To provide a more targeted approach for inhibiting inflammation, the receptors and early signaling events responsible for driving cytokine production need to be identified. Studies from both human and murine models of arthritis have indicated that the endosomal TLRs or their signaling pathways may offer potential targets for the development of new therapeutics for RA.

## Experimental Arthritis Models

One of the most commonly used methods of experimental arthritis is the murine collagen-induced arthritis (CIA) model. CIA has been observed to have numerous similarities to RA including erosive joint damage and production of circulating autoantibodies targeting type-II collagen (26). Initial studies utilizing knockout mice suggested roles for MyD88, TLR2, and TLR4 (27, 28). However, induction of CIA relies upon complete Freund’s adjuvant (CFA), which contains TLR2, 4, and 9 ligands, making interpretation of these results difficult. In recent years, research has expanded to the endosomal TLRs, with experiments being performed across a variety of models, including serum transfer models and the rat pristane-induced arthritis (PIA) model. These additional models also share pathological features of RA but unlike the CIA model they do not require CFA for disease induction.

In the rat PIA model, a role was proposed for TLR3 following the observations that TLR3 was significantly up-regulated in splenocytes following pristane injection and stimulation of TLR3 with poly-I:C exacerbated disease. Furthermore, in the same study, down-regulation of TLR3 with siRNA ameliorated disease, pointing to a key role in disease progression (29). Similarly, up-regulation of TLR3 has been observed in splenocytes from both PIA and CIA rat models, where administration of MTX inhibited both disease symptoms and the increase in TLR3 expression (30). Interestingly, synovial fibroblasts from PIA susceptible Dark Agouti rats show elevated levels of TLR3 following co-culture with conditioned media of pristane-stimulated T-cells (31), suggesting a system whereby immune-surveillant cells might prime synoviocytes toward TLR responses. Together, these data suggest that TLR3 may function in the maintenance of disease. However, a conflicting picture has emerged in the mouse CIA and K/BxN serum transfer models where activation of TLR3 suppressed arthritis, signifying a regulatory role in controlling rather than promoting inflammation (32).

More consistency has been observed across disease models for TLR7. Induction of TLR7 tolerance by a short-course treatment with a TLR7 agonist (1V136) at levels insufficient to induce cytokine production ameliorated disease in the K/BxN serum transfer model (33). However, additional cross tolerance of TLR2 and TLR9 was also observed, possibly due to activation of the negative regulators IRAK-M (interleukin-1 receptor-associated kinase-M) and SHIP-1 (Src homology 2 domain containing inositol polyphosphate phosphatase), making it difficult to delineate which TLR was responsible for the reduction in disease. Supportive of a pathological role for TLR7, we have demonstrated in the murine CIA model that TLR7^−/−^ mice show a reduction in clinical score, paw swelling, and number of paws affected when compared to wild type (WT) mice. This effect was limited to the maintenance of disease rather than the initial onset, as demonstrated by a similar number of WT and TLR7^−/−^ mice developing disease (34). Similarly, intra-articular knockdown of TLR7 using lentiviral delivery of TLR7 shRNA has been reported to decrease radiologic measures of disease activity in the rat CIA model (35).

The involvement of TLR8 in these models and the subsequent relevance of the data to human disease are less straightforward. Although TLRs 7 and 8 both recognize ssRNA and are closely phylogenetically related, in the murine system, only TLR7 is capable of activating downstream signaling in response to ssRNA, whereas in human cells both receptors are functional (36). TLR8^−/−^ mice develop spontaneous autoimmunity, characterized by increased serum levels of IgM, IgG2a, and autoantibodies against small nuclear ribonucleoproteins and dsDNA. Dendritic cells (DCs) from these mice had increased TLR7 levels and were hyper responsive to TLR7 activation. Concomitant TLR7 and TLR8 knockout, or individual TLR7 knockout mice did not exhibit this autoimmune phenotype, suggesting the potential for TLR8 to regulate TLR7 signaling in mice (37).

Similar to TLR3, TLR9 may have an anti-inflammatory role in arthritis. Numerous intracellular autoantigens, including self-nucleic acids, displayed on the cell surface of apoptotic cells (ACs) can activate an anti-inflammatory effect *in vivo* (38, 39). In the CIA model, administering ACs was shown to induce an anti-inflammatory effect dependent on the presence of TLR9, as determined using TLR9^−/−^ mice. In addition, this effect was abolished following DNAse treatment of ACs prior to treatment of CIA mice (39). Thus, TLR9 may function to induce tolerance and could have an important role in the prevention of inflammation. Indeed, TLR-induced type-I IFN has been suggested to be protective in a murine serum transfer model of arthritis. Protein tyrosine phosphatase non-receptor type 22 (PTPN22), a gene associated with RA susceptibility, has been shown to be required for IFN production upon TLR3, 4, 7, and 9 stimulation (40). PTPN22 knockout mice were observed to no longer exhibit disease suppression following TLR ligation in this arthritis model (41). This finding suggests that the type-I IFN response may explain the anti-inflammatory, tolerogenic role of TLR9 signaling. Interestingly, an association has been observed between RA susceptibility and a polymorphism in IRF5; a transcription factor downstream of the endosomal TLRs, involved in the production of IFN (42). However, the role of IFN currently remains controversial; whilst administration of IFN-β can ameliorate disease in the murine CIA model, a double-blind clinical trial found no significant improvement between IFN-β and placebo-treated groups (43, 44).

Conversely, inhibition of TLR9 with an immunoregulatory DNA sequence has been reported to alleviate disease activity and delay disease onset in the rat PIA model, indicating a potential role for TLR9, and possibly DNA ligands, in the development of disease (45). Indeed, DNAse-II deficient mice develop an RA-like polyarthritis, however, this was not dependent on TLR9, as arthritis was also observed in DNAse-II/TLR9 double knockout mice, suggesting that other innate DNA sensors may be of more importance (46).

Though the duality of the effects of TLR stimulation in RA models may appear contradictory, these observations may in fact provide insights into fundamental TLR biology. Under some conditions, TLR signaling may be immunoregulatory, however in the presence of elevated levels of DAMPs and accessory molecules such as LL-37, this may lead to pathological inflammation given a susceptible genetic background (47). Similar complexity has been reported in murine models of the IFN-mediated autoimmune disease, systemic lupus erythematosus (SLE). An immunoregulatory role was attributed to TLR9 following knockout, despite its requirement for pathogenic anti-DNA autoantibody production (48). However, inhibition of TLR9 has indicated a pro-inflammatory role in the production of IFN in response to DNA containing immune complexes (49).

Despite the seemingly conflicting evidence for the involvement of some of the endosomal TLRs in experimental arthritis models, overall it would appear that there may be a role for one or more of these receptors in driving the disease process. In fact, we have demonstrated that combined inhibition of these receptors using an off target effect of some anti-depressant drugs, therapeutically reduced clinical score, paw swelling, and histological joint damage in the murine CIA model (34, 50). Furthermore, it has been reported that activation of TLR7 and TLR9 with heterogeneous nuclear ribonucleoprotein (hnRNP) antigens from splenocytes of a PIA animal can be used to transfer disease to another animal. Nuclease treatment of hnRNPs or treatment with hnRNP-associated nucleic acids alone demonstrated that only the nucleic acid components were required (51). Interestingly, humoral and cellular autoimmunity to hnRNP-A2/B1 is present in approximately 50% of RA patients, which may be linked with the ability of hnRNPs to activate TLR7 and TLR9 (51).

## Endosomal TLRs in RA

The potential role of TLRs in RA has led to a number of genetic studies, including a genome-wide association study examining common TLR single nucleotide polymorphisms (SNP). However, no significant associations with RA susceptibility, severity, or response to treatment have been identified (52–54). However, some small studies have revealed possible associations with TLR SNPs in distinct ethnic cohorts. The TLR9 SNP rs187084 allele variant TT has been modestly associated with RA susceptibility in a Turkish RA population, although it was not linked in studies of Dutch or French cohorts (53–55). Interestingly, the TLR8 SNP rs5741883 has been associated with rheumatoid factor autoantibody positivity in one study of a European population (56). Nonetheless, functional studies of the endosomal TLRs in human samples have suggested the potential involvement of these receptors and their signaling pathways in RA. Indeed, we previously demonstrated a role for MyD88, the adaptor protein used by TLRs 7, 8, and 9, in the spontaneous release of cytokines and MMPs from human RA synovial tissue cultures (57).

Many studies have examined the expression and function of these receptors in synovial tissue cells from patients compared with tissue from osteoarthritis (OA) patients or healthy controls (HCs). TLRs 3, 7, 8, and 9 are all expressed in the RA synovium, which is a mixed cell population (58–60), with levels of TLRs 3, 7, and 9 elevated in comparison to either OA or HC tissue, particularly in RASFs, DCs, and macrophages (59–62). Stimulation of RA synovial cells with ligands for TLR3 and TLR8 can induce production of cytokines and MMPs, indicating that these TLRs are functional within RA joint tissue (58, 60). In fact, out of a range of TLR ligands, TLR8 induced the biggest secretion of TNF from RA synovial cultures (58). Interestingly, mimicking the hypoxic conditions of the *in situ* joint has been shown to exacerbate cytokine and MMP production following activation of TLR3 in RASFs (63). This elevated response to hypoxia may support pathogenic angiogenesis in the synovium through the ability of TLR3 to induce vascular endothelial growth factor and IL-8 (64). Furthermore, TLR3 can also promote osteoclastogenesis both directly and indirectly via up-regulation of RANKL on RASFs following activation (65).

For endosomal TLRs to be activated in the RA joint, suitable ligands would need to be present. RA necrotic synovial fluid cells have been shown to release RNA that can activate TLR3 on RASFs (59). Additionally, DNA released from necrotic cells may induce cytokine production in a TLR9-dependent manner (59, 62, 66). Nucleic acids are readily degraded outside of the cell, however in the RA joint they may be protected by other molecules. The cathelicidin family member human cationic antimicrobial peptide LL-37 is present in RA synovial fluid at levels greater than HCs and is known to bind RNA, protecting it from degradation (47, 67). In addition, LL-37 is known to perpetuate signaling through TLRs 3, 7, 8, and 9 (67–69).

Further support for a contribution of these TLRs to the perpetuation of inflammation in RA comes from the antimalarial drugs chloroquine, hydroxychloroquine, and quinacrine, which have been used since 1950s to treat RA and SLE (70, 71). These drugs act as antagonists of TLR9 and to a lesser extent, TLR3, TLR7, and TLR8 (72, 73). The mechanism was suggested to be due to the inhibition of endosomal acidification; however, more recently antimalarials have been shown to interact directly with nucleic acids, causing modifications which prevent their binding to the endosomal TLRs (73). We found that, when added to human RA synovial cultures, chloroquine suppressed spontaneous cytokine production, indicating the possibility of nucleic acids driving inflammation in these samples (58). In addition to these results, the anti-depressant inhibitors of the endosomal TLRs that were effective in the CIA model also suppressed spontaneous cytokine production in human RA synovial membrane cultures (34, 50, 58). Neither of these approaches represent a clinically useful therapy for RA, due to the effective dose exceeding clinically safe levels. However, these studies, as summarized in Table 1, do highlight a possible role for endosomal TLRs in the maintenance of inflammation, as well as demonstrating the potential to selectively target these receptors for the effective treatment of RA.

**Table 1:** **Dual nature of endosomal TLR signaling in RA and disease models**.

	Immunoregulatory	Immunostimulatory
TLR3	Activation of TLR3 in CIA and K/BxN serum transfer models suppresses arthritis (32)	Activation of TLR3 exacerbates PIA. In agreement, knockdown of TLR3 ameliorated disease (29)
		Stimulation of TLR3 induces angiogenic and osteoclastogenic factors in human RA synovial fibroblasts (64, 65)
		Hypoxia induces heightened responses from TLR3 in RASFs (63)
TLR7		TLR7 has been suggested to contribute to CIA pathology in established disease (34)
		hnRNP can activate TLR7 and can transfer disease in the PIA model (51)
		TLR7 tolerance induced by sub-optimal stimulation alleviates the K/BxN serum transfer model (33)
TLR8	TLR8 has a potential role in suppressing TLR7 responses in murine models (37)	In human RA synovial cultures, stimulation of TLR8 results in the greatest cytokine production (58)
		Inhibition of TLR8 inhibits spontaneous cytokine release in a human RA synovial tissue model (58, 74)
TLR9	An anti-inflammatory response is induced on exposure of TLR9 to apoptotic cells (55)	Inhibition of TLR9 alleviates rat PIA model (45)
		hnRNP acts as a ligand for TLR9 and can transfer disease in the PIA model (51)

*Seemingly contradictory findings regarding the roles of the endosomal TLRs in human RA tissue models and differing experimental arthritis models have been identified*.

## Targeting the Endosomal TLRs Therapeutically

There has been significant interest in the modulation of TLR function in disease. In particular, endosomal TLR antagonists are in development as promising candidates for the treatment of several autoimmune diseases. Indeed, some therapies already in the clinic are suggested to mediate their effect through endosomal TLR blockade. As already mentioned, antimalarials are used for the treatment of RA, but at the doses given clinically they will only deliver a moderate effect on disease and are thus usually administered in combination with other therapies (75). This is most likely due to the serum levels not reaching those required for effective TLR inhibition. Similarly, the gold-containing complex, auranofin, has also been suggested to moderate its’ anti-rheumatic effect through blockade of TLR3-dependent TRIF signaling (76).

With the aim of designing more effective therapies specifically targeting TLRs in autoimmunity, many small molecules and immune response modifiers are under development. A small molecular weight inhibitor, CPG-52364, was initially described for the treatment of SLE, but may have therapeutic potential in other autoimmune diseases such as RA and psoriasis. CPG-52364 inhibits signaling following stimulation of TLR7, 8, or 9 in human PBMCs (77); CPG-52364 entered Phase I clinical development in 2007, however, the results of this trial are yet to be published.

Other inhibitors have followed into the clinical trial arena. Dynavax Technologies demonstrated, in both plasmacytoid DCs from SLE patients and in two lupus-prone mouse strains, that glucocorticoid resistance could be mediated through activation of TLR7 and TLR9 (78). Glucocorticoids are frequently used for the treatment of many autoimmune and inflammatory conditions, including RA, but the high doses required for effective treatment lead to significant side-effects and restrict use. Dynavax Technologies in partnership with GlaxoSmithKline have consequently developed DV1179, a bifunctional inhibitor of TLR7 and TLR9, which has been shown to reverse glucocorticoid resistance in both human cells and animal models of lupus. DV1179 was well-tolerated in a Phase I clinical trial and subsequently, a proof of mechanism clinical study was initiated in SLE patients in late 2011 (79).

Idera Pharmaceuticals has two lead candidates in development for the treatment of autoimmune diseases; IMO-3100, an antagonist of TLRs 7 and 9 and IMO-8400, an antagonist of TLRs 7, 8, and 9. In preclinical models of autoimmune diseases including CIA, lupus, and psoriasis, these TLR antagonists have been shown to inhibit Th1, Th17, and inflammasome pathways and suppress production of cytokines such as TNF, IL-12, IL-6, and IL-17 (80, 81). The results of a Phase II clinical trial were recently announced for IMO-3100; Idera reported that clinical improvements observed in psoriasis patients treated with IMO-3100 for 4 weeks correlated with the proposed mechanism of action for TLR antagonism (80). IMO-8400 entered a Phase I clinical trial in late 2012 to assess safety in healthy subjects. VentiRx Pharmaceuticals is also developing a TLR8 specific antagonist for the treatment of autoimmune diseases; VTX-763 is a preclinical lead which shows marked inhibition of TLR8-induced NF-κB activation and TNF production *in vitro* (74).

## Conclusion

The advent of anti-TNF therapeutics for the treatment of RA has not only resulted in greatly enhanced disease outcomes for many patients but has also raised a number of new questions regarding the production of TNF, and the role of the innate immune system in RA pathogenesis. Activation of endosomal TLRs represents a potential mechanism, which may contribute to the cytokine production characteristic of the RA synovium. Key roles have been attributed to these receptors in both murine and human models of disease. Accordingly, numerous early-stage therapeutics targeting endosomal TLRs and their signaling networks are in development. Concerns will always be raised over targeting molecules with key roles within the immune system. However, with a diverse range of innate immune receptors able to detect viral and bacterial pathogens, it may be possible to target the endosomal TLRs without compromising innate immunity due to compensation from the other cytosolic RNA and DNA receptors such as RIG-I (retinoic acid inducible gene-I) and AIM2 (absent in melanoma 2) (5). The desired therapy would provide a small molecular weight inhibitor to deliver ease of administration with a lower production cost than current biological therapies. It is also hoped that targeting upstream disease mechanisms may additionally afford greater efficacy than global suppression of individual cytokines.

## Conflict of Interest Statement

The authors declare that the research was conducted in the absence of any commercial or financial relationships that could be construed as a potential conflict of interest.

## Abbreviations

AC, apoptotic cell; CFA, complete Freund’s adjuvant; CIA, collagen induced arthritis; CTLA-4, cytotoxic T-lymphocyte-associated antigen 4; DAMPs, damage associated molecular patterns; DC, dendritic cell; DMARD, disease-modifying anti-rheumatic drug; HMGB-1, high mobility group box 1; hnRNP, heterogenous nuclear ribonucleoprotein; IFN, interferon; IRF, interferon regulatory factor; LL-37, cathelicidin family member human cationic antimicrobial peptide LL-37; LPS, lipopolysaccharide; MAL, MyD88 adaptor like; MMP, matrix metalloprotease; MTX, methotrexate; MyD88, myeloid differentiation primary response 88; PIA, pristane induced arthritis; PTPN22, protein tyrosine phosphatase non-receptor type 22; RA, rheumatoid arthritis; RANKL, receptor activator of nuclear factor κB ligand; RASF, rheumatoid arthritis synovial fibroblast; SLE, systemic lupus erythematosus; TLR, toll-like receptor; TNF, tumor necrosis factor; TRAM, TIR domain containing adaptor molecule 2; TRIF, TIR domain containing adaptor inducing interferon-β; WT, wild type.
